# Timing of Lung Transplant Referral in Patients with Severe COVID-19 Lung Injury Supported by ECMO

**DOI:** 10.3390/jcm12124041

**Published:** 2023-06-14

**Authors:** Liran Levy, Ofir Deri, Ella Huszti, Eyal Nachum, Stephane Ledot, Nir Shimoni, Milton Saute, Leonid Sternik, Ran Kremer, Yigal Kassif, Nona Zeitlin, Jonathan Frogel, Ilya Lambrikov, Ilia Matskovski, Sumit Chatterji, Lior Seluk, Nadav Furie, Inbal Shafran, Ronen Mass, Amir Onn, Ehud Raanani, Amir Grinberg, Yuval Levy, Arnon Afek, Yitshak Kreiss, Alexander Kogan

**Affiliations:** 1 The Sheba Lung Transplant Program, Sheba Medical Center, Sackler School of Medicine, Tel Aviv University, Tel Aviv 69978, Israel; 2 Institute of Pulmonary Medicine, Sheba Medical Center, Sackler School of Medicine, Tel Aviv University, Tel Aviv 69978, Israel; 3 Biostatistics Research Unit, University Health Network, Toronto, ON M5G 1X6, Canada; 4 Department of Cardiac Surgery, Leviev Cardiothoracic and Vascular Center, Sheba Medical Center, Tel Hashomer, Sackler School of Medicine, Tel Aviv University, Tel Aviv 69978, Israel; 5 Department of Anesthesiology, Sheba Medical Center, Tel Hashomer, Sackler School of Medicine, Tel Aviv University, Tel Aviv 69978, Israel; 6 Department of Thoracic Surgery, Leviev Cardiothoracic and Vascular Center, Sheba Medical Center, Tel Hashomer, Sackler School of Medicine, Tel Aviv University, Tel Aviv 69978, Israel; 7 General Management, Sheba Medical Center, Tel Hashomer, Sackler School of Medicine, Tel Aviv University, Tel Aviv 69978, Israel

**Keywords:** lung transplant, coronavirus disease 2019 (COVID-19), acute respiratory distress syndrome (ARDS), extracorporeal membrane oxygenation (ECMO)

## Abstract

Severe respiratory failure caused by COVID-19 often requires mechanical ventilation, including extracorporeal membrane oxygenation (ECMO). In rare cases, lung transplantation (LTx) may be considered as a last resort. However, uncertainties remain about patient selection and optimal timing for referral and listing. This retrospective study analyzed patients with severe COVID-19 who were supported by veno-venous ECMO and listed for LTx between July 2020 and June 2022. Out of the 20 patients in the study population, four who underwent LTx were excluded. The clinical characteristics of the remaining 16 patients were compared, including nine who recovered and seven who died while awaiting LTx. The median duration from hospitalization to listing was 85.5 days, and the median duration on the waitlist was 25.5 days. Younger age was significantly associated with a higher likelihood of recovery without LTx after a median of 59 days on ECMO, compared to those who died at a median of 99 days. In patients with severe COVID-19-induced lung damage supported by ECMO, referral to LTx should be delayed for 8–10 weeks after ECMO initiation, particularly for younger patients who have a higher probability of spontaneous recovery and may not require LTx.

## 1. Introduction

The emergence of coronavirus disease 2019 (COVID-19), caused by the novel severe acute respiratory syndrome coronavirus 2 (SARS-CoV-2), has caused significant morbidity and mortality worldwide [1,2]. As the pandemic continues to ravage communities, the scientific community has been working tirelessly to better understand the virus and its impact on human health.

One of the most devastating consequences of COVID-19 is its detrimental effect on the lungs, which is evident in a wide range of clinical manifestations varying from mild respiratory symptoms to severe respiratory disease. Approximately 6% to 10% of individuals with COVID-19 may develop acute respiratory distress syndrome (ARDS) [3,4], which often requires mechanical ventilation and/or extracorporeal membrane oxygenation (ECMO). In exceptional circumstances, a lung transplant has been proposed as a potential solution for COVID-19 ARDS patients who do not show improvement [5,6,7]; however, data are still lacking to inform selection criteria for potential recipients, the optimal timing of referral or listing, and long-term outcomes.

As one of only two lung transplant programs in Israel, our program has been at the forefront of managing severe COVID-19-related lung injury. Since the onset of the pandemic, patients with severe lung injury who were supported by prolonged ECMO with the goal of lung transplantation were exclusively referred to and listed at our center. However, due to the limited availability of donor lungs and the prolonged waitlist period, only a small proportion of patients were able to undergo a lung transplant, while others either died or experienced unexpected recovery. This unique situation has allowed us to gain valuable insights into the potential clinical outcomes for the most severe COVID-19 patients on ECMO support.

The current study aimed to retrospectively review the clinical characteristics and outcomes of patients with severe COVID-19-related lung injury supported by ECMO who were listed for a lung transplant and determine parameters associated with recovery versus death. Our experience may add to the evolving data on patient selection and timing of referral and listing of this patient population.

## 2. Methods

### 2.1. Study Design and Patient Population

This study presents a retrospective case series review that includes an analysis of all adult patients who were referred to the Sheba Medical Center for a lung transplant due to severe COVID-19-related lung injury. The Sheba lung transplant program was launched in December 2020 and has been performing around 10–20 transplants a year. The study period spanned from 1 July 2020 to 30 June 2022, and the research was approved by the institutional research ethics board, ensuring compliance with ethical guidelines. Follow-up data were obtained from electronic medical records and computerized databases, and the analysis was censored on 30 June 2022.

### 2.2. Referral Process

Referral for lung transplant was typically made by an intensive care unit specialist or a pulmonologist. All referred patients were assessed by one of the transplant physicians or intensive care unit staff in the referring center. This evaluation encompassed a comprehensive assessment of the patient’s medical history, a thorough physical examination, and a thorough review of laboratory tests and imaging studies. The reasons for referral were discussed in detail with the patient’s relatives, and if possible, with the patients themselves, taking into consideration their preferences and input.

### 2.3. Evaluation for Lung Transplant

Patients were considered eligible for a lung transplant in case they had shown no longitudinal evidence of lung recovery (i.e., radiological evidence of irreversible lung disease affecting all lobes with no radiological or clinical improvement despite mechanical ventilation, ECMO support, or a combination of mechanical ventilation and ECMO) after at least 4 to 6 weeks had elapsed from the onset of COVID-19-related ARDS in accordance with published expert opinion and guidance from the International Society of Heart and Lung Transplantation [8,9]. Additional criteria included: (1) younger than 65 years; (2) negative for SARS-CoV-2 on specimens obtained by endotracheal aspirate or bronchoscopy 24 h apart; (3) single organ dysfunction; (4) no evidence of irrecoverable brain damage; and (5) fulfilled the remaining typical criteria for lung transplant [10]. Patients with left heart failure, significant coronary disease, obesity (body mass index > 35 kg/m^2^), active or recent malignancy, and significant liver injury were excluded. Patients with a combination of relative contraindications such as diabetes, hypertension, and other co-morbidities were also excluded. The final decision to transfer a patient and place them on the lung transplant list was made by a multidisciplinary team after careful evaluation and deliberation, taking into account various factors, including the patient’s clinical condition, prognosis, and overall suitability for a lung transplant.

### 2.4. Patient Care in the Intensive Care Unit

All listed patients were admitted to the cardiac surgery intensive care unit and received treatment according to the local standard of care involving a multidisciplinary team that included transplant surgeons, pulmonologists, critical care physicians, infectious disease physicians, dieticians, physiotherapists, social workers, and intensive care unit nurses. Patients were bridged with ECMO or a combination of mechanical ventilation and ECMO. ECMO was performed with a Permanent Life Support or Cardiohelp HLS module 7.0 (Maquet, Rastatt, Germany) using percutaneous or surgical access to jugular, subclavian or femoral arteries (17-21-F) or veins (21-29-F). Unfractionated heparin was administrated during ECMO with a partial thromboplastin time between 60 and 80 s. Patients were preferentially awake during bridging so they could be physically active daily and involved in the decision-making process. If awake bridging was not feasible, daily sedation holds were performed in line with critical care best practices. Lung recovery was defined as successful weaning and decannulation from ECMO, followed by discharge from the intensive care unit.

### 2.5. Statistical Analysis

Demographic characteristics were assessed as counts and percentages for categorical variables and as standard measures (median and interquartile range (IQR)) for continuous variables. To compare baseline characteristics between groups, we used the Wilcoxon rank-sum test for continuous variables and Fisher exact test for categorical variables. The date of discharge from the intensive care unit was used as the date of recovery. All analyses were conducted using R statistical software version 3.4.3 (R Foundation for Statistical Computing, Vienna, Austria). Statistical significance was defined as a two-tailed *p* value less than or equal to 0.05.

## 3. Results

### 3.1. Study Population and Patient Characteristics

**Figure 1:**
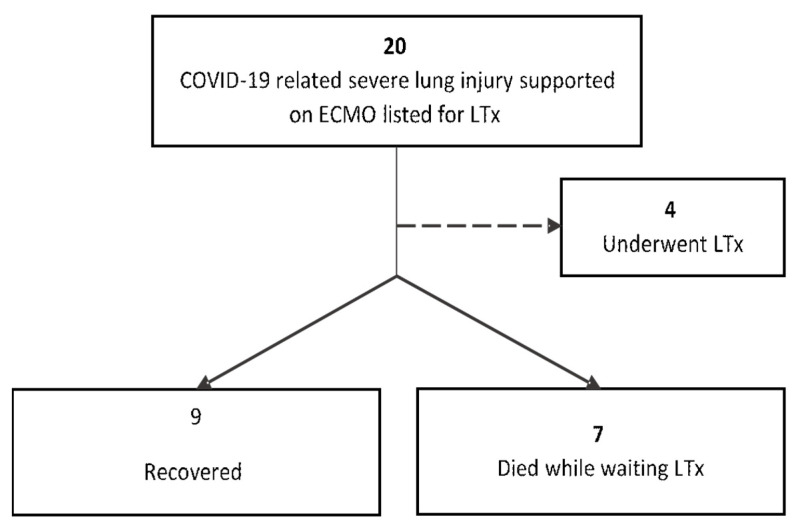
CONSORT flow diagram.

Over the course of the study period, 20 patients with severe COVID-19-related lung injury supported on ECMO were referred and listed for a lung transplant. The median time on the waitlist was 25.5 days (IQR 14.5–31.5). Of the total study cohort, 4 (20%) underwent lung transplant, 9 (45%) achieved spontaneous recovery, and 7 (35%) died while awaiting transplant. All recovered patients were discharged from the ICU and remained alive without requiring a lung transplant at the time of writing this report. All patients who died experienced sepsis as the cause of death. A CONSORT flow diagram is depicted in Figure 1.

**Figure 2:**
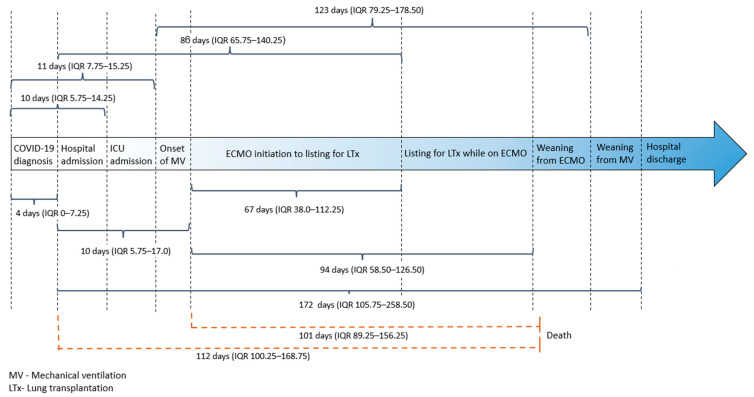
Overall time intervals (median and IQR).

The study cohort comprised individuals with a median age of 49.5 years (interquartile range (IQR): 43.8, 57.5) and a male preponderance (60%). The median body mass index at admission was 30.5 (IQR: 28.9, 31), and obesity was the most common comorbidity, affecting 18 patients (90%). Other comorbidities, such as dyslipidemia, hypertension, diabetes, and heart or lung disease, were less frequent. A history of tobacco use was noted in 2 patients (10%), and only one patient had a remote history of cancer. All patients received invasive mechanical ventilation and venovenous extracorporeal membrane oxygenation during their intensive care unit stay. The median time from COVID-19 diagnosis to hospital admission was 4 days (IQR: 0, 7.25). The median time from hospital admission to ECMO initiation was 10 days (IQR: 5.75, 17), and the median time from hospital admission to lung transplant listing was 86 days (IQR: 65.75, 140.25). The median time from hospital admission to death was 112 days (IQR: 100.25, 168.75), and from ECMO initiation to death, 101 days (IQR: 89.25, 156.25). The clinical characteristics of the study group are summarized in Table 1, Table S1 and Figure 2.

### 3.2. Clinical Outcomes

Our study aimed to compare the clinical characteristics of patients on the lung transplant waitlist who spontaneously recovered and those who died, with the goal of identifying potential parameters associated with their respective outcomes. Our results revealed significant differences in age and duration of ECMO support between the two groups. Notably, patients who recovered had a median age of 44 years (IQR: 41, 55), while those who died had a median age of 61 years (IQR: 49.5, 65.5), *p* value = 0.016. Additionally, the duration of ECMO support was significantly shorter in the recovery group, with a median of 59 days (IQR: 53, 93), compared to 99 days (IQR: 84, 138) for those who died, *p* value = 0.044. Interestingly, we found no significant differences in the incidence of comorbidities, body mass index, or other time intervals between the two groups. Furthermore, none of the patients who recovered had received the COVID-19 vaccine, while only one patient in the group who died had been vaccinated. The detailed findings are presented in Table 1 and Table 2, as well as in Tables S1 and S2 in the Supplementary Materials.

## 4. Discussion

This retrospective study provides a detailed analysis of the clinical characteristics and outcomes of patients who experienced severe COVID-19-related lung injury and were bridged to lung transplant using ECMO support. The study took advantage of the extended local waitlist times and the ability to provide ECMO support for longer durations, allowing for meticulous observation of the disease course in these patients. The findings revealed that younger patients had a higher likelihood of recovery without the need for a lung transplant, with ECMO weaning occurring approximately two months after initiation. This insight contributes to the growing body of evidence regarding optimal lung transplant selection criteria and timing. It aids in informed decision making to maximize the benefits of a lung transplant while avoiding premature consideration, which can potentially improve patient outcomes.

Although lung transplant is a viable option for a variety of end-stage lung diseases, prior to the COVID-19 pandemic, patients with acute lung injury due to infectious causes were infrequently considered for transplantation [11]. The COVID-19 pandemic, with its catastrophic lung injury, has brought to attention lung transplant as a salvage therapy for patients that are unlikely to recover despite maximum ventilatory support, the use of ECMO, and optimal medical care. The safety and outcomes of lung transplants for these patients have been confirmed in several publications that report short-term post-transplant survival comparable to non-COVID-19-related ARDS lung transplants [12]. A study published by Kurihara et al. in the *Journal of the American Medical Association* reported that lung transplant recipients with COVID-19-related ARDS had similar short-term outcomes compared to non-COVID-19-related ARDS recipients. The study followed 102 lung transplant recipients, including 30 with COVID-19-related ARDS [12]. Another study published in the *New England Journal of Medicine* in 2021 reported similar findings. The study included 214 lung transplant recipients with COVID-19-related severe lung injury, with a 30-day mortality of 2.2% and a 3-month survival rate of 95.6% [13]. Overall, these studies offer significant evidence that supports the safety and effectiveness of lung transplants as a treatment option for patients with severe COVID-19-related ARDS. However, questions regarding patient selection arise due to uncertainties about the clinical course of lung disease and recovery, especially in terms of the likelihood of pulmonary functional improvement after prolonged ECMO support.

A limited number of studies have attempted to explore the effective management of severe COVID-19-induced ARDS, including the appropriate timing for referring patients for a lung transplant. King and colleagues introduce the general concept of a “transplant sweet spot”, which refers to a period when patients are unlikely to recover from their illness but have not yet experienced severe complications or deconditioning that would preclude transplantation [14]. In contrast, Cypel and colleagues strive for greater accuracy by proposing a specific time frame of four weeks of ECMO support for assessing the suitability of COVID-19-induced ARDS patients for lung transplants [9]. Yet, there is insufficient data to support and establish the optimal window for transplantation in this setup. Filling this gap, the present study offers a unique opportunity to investigate the different approaches and suggests that delaying lung transplant listing for 8–10 weeks may be beneficial, particularly for younger patients who are more likely to recover without requiring lung transplant operation.

Our study contributes to the existing knowledge on the use of ECMO as a bridge to lung transplant or recovery. Despite the risks associated with ECMO [15], it is believed to be beneficial in reducing mortality rates in carefully selected patients who receive treatment at specialized ECMO centers. One area of ongoing debate is determining the appropriate timing to switch from a bridge-to-recovery strategy to a bridge-to-transplant approach in cases of irreversible respiratory failure [15,16,17]. Several recent studies have indicated that prolonged ECMO support can allow for recovery of the native lung without the need for a lung transplant, and this approach has not been associated with adverse outcomes [18,19,20,21,22]. Our findings align with this approach and provide further support for the concept of native lung recovery following prolonged ECMO support. We have demonstrated in this study cases where patients who were initially deemed to have irreversible respiratory failure were able to recover their native lung function to such an extent that they could be successfully weaned off ECMO and mechanical ventilation. This suggests that a lung transplant may not be necessary for all patients, and a bridge-to-recovery strategy can be successful in selected cases. Our study underscores the importance of careful patient selection and individualized management strategies for patients with COVID-19 severe respiratory failure.

In the current work, age emerged as a significant factor associated with survival in patients with COVID-19-related ARDS. This finding echoes previous research on COVID-19 and ARDS, as older age has consistently been associated with a greater risk of death in COVID-19 patients, regardless of their candidacy for a lung transplant [23]. Furthermore, a similar phenomenon has also been reported in non-COVID-19 ARDS [24,25], suggesting that advanced age may be a significant risk factor for mortality in critically ill patients, regardless of the underlying cause of ARDS. This could be attributed to reduced physiological reserve in older patients, which may not become apparent until they are exposed to critical illness stressors. Although increased mortality with advanced age may also be related to the occurrence of multiple comorbid conditions, we did not find differences in the number or type of comorbidities between patients who died and those who recovered in our cohort. 

The strength of this study lies in its unique opportunity to track the natural history of patients who were considered to have severe COVID-19-related lung injury deemed irreversible and subsequently referred and listed for a lung transplant. However, it is important to interpret the findings in light of several limitations. Firstly, the retrospective nature of the study conducted in a single, highly experienced ECMO center (an annual volume of 100–150 ECMO runs, with approximately 50% of them being in the VV configuration) may limit the generalizability of the results to other settings. Yet, retrospective studies can still provide valuable insights and generate hypotheses for further investigation. This study serves as an initial exploration of patient selection and timing for referral and listing in the context of severe COVID-19 and ECMO support. Secondly, the small sample size used in this study should be taken into consideration when interpreting the findings, as it may impact the statistical power and precision of the results. Thirdly, the study did not assess the factors related to the management of patients in different hospitals prior to their transfer to the intensive care unit, which could potentially have influenced outcomes. Lastly, it should be noted that the highly selected nature of the study population, consisting of patients referred for a lung transplant, may have contributed to the relatively low number of significant differences observed between survivors and non-survivors. Overall, while the current study has its limitations, it serves as a starting point for further investigation in this important area of research. The findings should be interpreted with caution, and future studies can build upon these preliminary findings to provide more robust and meaningful conclusions.

## 5. Conclusions

In summary, the findings of this study provide valuable insights into the optimal timing for considering a lung transplant in patients with severe lung injury related to COVID-19. It is recommended to delay lung transplant consideration for approximately 8–10 weeks from the initiation of ECMO, especially in younger populations, as their lungs may have the potential for unexpected regenerative capacity and recovery.

## Figures and Tables

**Table 1:** Clinical and demographic characteristics of the study cohort and patients who recovered vs. died while waiting for lung transplant.

	Overall	Recovered While Awaiting Transplant	Died While Awaiting Transplant	*p* Value
N	20	9	7	
Gender = male (%)	12 (60.0)	6 (66.7)	5 (71.4)	0.999
Age (median (IQR))	49.5 [43.8, 57.5]	44.0 [41.0, 55.0]	61.0 [49.5, 65.5]	0.016
BMI at admission (median (IQR))	30.5 [28.9, 31.0]	30.4 [28.8, 31.2]	30.8 [28.4, 30.9]	0.999
Prior comorbidities, n (%)				
Cardiovascular disease	1 (5.0)	1 (11.1)	0 (0.0)	0.999
HTN	5 (25.0)	1 (11.1)	2 (28.6)	0.809
Diabetes	3 (15.0)	0 (0.0)	1 (14.3)	0.896
Obesity	18 (90.0)	8 (88.9)	6 (85.7)	0.999
Dyslipidemia	7 (35.0)	4 (44.4)	1 (14.3)	0.455
Smoking Hx	2 (10.0)	1 (11.1)	0 (0.0)	0.999

Abbreviations: BMI = body mass index; HTN = hypertension.

**Table 2:** Differences in time intervals between patients who recovered vs. died while waiting for lung transplant.

Time Intervals (Median (IQR))	Recovered While Awaiting Transplant	Died While Awaiting Transplant	*p* Value
Time from COVID-19 to hospital admission	4.0 [0.0, 7.0]	5.0 [0.5, 10.5]	0.384
Time from COVID-19 to MV	10.0 [9.0, 15.0]	12.0 [4.0, 21.0]	0.873
Time from COVID-19 to ECMO	15.0 [13.0, 30.0]	12.0 [7.0, 26.0]	0.289
Time from COVID-19 to listing	87.0 [65.0, 98.0]	79.0 [52.5, 116.0]	0.672
Time from ECMO to listing	56.0 [32.0, 68.0]	68.0 [44.0, 103.0]	0.368
Time on MV	142.0 [58.0, 222.0]	101.0 [90.0, 140.0]	0.958
Time on ECMO	59.0 [53.0, 93.0]	99.0 [84.0, 138.0]	0.044
Time on Tx list	20.0 [16.0, 25.0]	29.0 [21.0, 53.0]	0.186
Time from COVID-19 to death	--	115.0 [106.0, 147.5]	--

Abbreviations: COVID = coronavirus disease 2019; ECMO = extracorporeal membrane oxygenation; MV = mechanical ventilation; Tx = transplant.

## Data Availability

The data presented in this study are available on request from the corresponding author. The data are not publicly available due to privacy restrictions.

## Supplementary Materials

The following supporting information can be downloaded at: https://www.mdpi.com/article/10.3390/jcm12124041/s1, Table S1: Additional clinical and demographic characteristics of the study cohort and patients who recovered vs. died while awaiting lung transplant; Table S2: Overall time intervals and differences in time intervals between patients who recovered vs. died while awaiting lung transplant.
