# Supplementary material for: Timing of Lung Transplant Referral in Patients with Severe COVID-19 Lung Injury Supported by ECMO
Source: J Clin Med. 2023 Jun 14;12(12):4041. doi: 10.3390/jcm12124041 (PMC10299376; doi:10.3390/jcm12124041)
Supplement: Supplementary file 1 [file jcm-12-04041-s001.zip › jcm-2359548-supplementary.pdf]

Table S1. Additional clinical and demographic characteristics of the study cohort and patients who recovered vs. died while awaiting a lung transplant

	Overall*	Recovered while awaiting transplant	Died while awaiting transplant	P value
n	20	9	7	
Gender, male (%)	12 (60.0)	6 (66.7)	5 (71.4)	0.99
Age (median [IQR])	49.50 [43.80, 57.50]	44.00 [41.00, 55.00]	61.00 [49.50, 65.50]	0.016
Weight at admission (median [IQR])	85.50 [80.00, 98.50]	90.00 [80.00, 100.00]	86.00 [78.50, 97.50]	0.671
Weight at discharge from ICU (median [IQR])	70.50 [68.50, 85.05]	70.00 [68.00, 90.50]	--	--
BMI at admission (median [IQR])	30.50 [28.87, 31.05]	30.40 [28.80, 31.20]	30.80 [28.45, 30.95]	0.999
BMI at discharge (median [IQR])	26.50 [24.42, 27.48]	26.40 [22.85, 29.20]	--	--
Transferred from another hospital (%)	16 (80.0)	6 (66.7)	6 (85.7)	0.771
Vaccinated (%)	1 (5.0)	0 (0.0)	1 (14.3)	0.896
**Patient's outcomes**				
Deceased (%)	8 (40.0)	0 (0.0)	7 (100.0)	--
Discharged from ICU (%)	10 (50.0)	7 (77.8)	0 (0.0)	0.009
Discharged from hospital (%)	9 (45.0)	7 (77.8)	0 (0.0)	0.009
Need of a nursing facility (%)	4 (36.4)	2 (25.0)	0 (NaN)	--
Discharged home (%)	9 (45.0)	7 (77.8)	0 (0.0)	0.009
Oxygen dependent (%)	9 (75.0)	7 (77.8)	0 (NaN)	--
**ECMO characteristics**				
Tracheostomy prior listing (%)	20 (100.0)	9 (100.0)	7 (100.0)	--
VV ECMO (%)	19 (95.0)	9 (100.0)	7 (100.0)	--
VA ECMO (%)	2 (10.0)	9 (100.0)	7 (100.0)	--
Awake ECMO (%)	6 (30.0)	3 (33.3)	1 (14.3)	0.771
Technical failure during ECMO support (%)	6 (30.0)	2 (22.2)	2 (28.6)	0.999
**Comorbidities during admission**				
Acute kidney injury (%)	8 (40.0)	3 (33.3)	3 (42.9)	0.999
Intracranial hemorrhage (%)	0 (0.0)	0 (0.0)	0 (0.0)	--
Other hemorrhage (%)	14 (70.0)	7 (77.8)	3 (42.9)	0.362

Liver failure (%)	7 (35.0)	2 (22.2)	4 (57.1)	0.362
Anemia (%)	20 (100.0)	9 (100.0)	7 (100.0)	--
Leukopenia (%)	0 (0.0)	0 (0.0)	0 (0.0)	--
Thrombocytopenia (%)	17 (85.0)	7 (77.8)	6 (85.7)	1
Heparin-induced thrombocytopenia (%)	5 (25.0)	1 (11.1)	2 (28.6)	0.809
Cardiac ischemia (%)	0 (0.0)	0 (0.0)	0 (0.0)	--
Heart failure with reduced EF (%)	1 (5.0)	9 (100.0)	7 (100.0)	--
Delirium other neurological sequela (%)	5 (25.0)	2 (22.2)	1 (14.3)	1
VTE (DVT/ PE) (%)	3 (15.0)	9 (100.0)	7 (100.0)	--
Stroke (%)	0 (0.0)	0 (0.0)	0 (0.0)	--
DIC (%)	0 (0.0)	0 (0.0)	0 (0.0)	--
Hemolysis (%)	3 (15.0)	1 (11.1)	1 (14.3)	0.999
SEPSIS during hospitalization (%)	20 (100.0)	9 (100.0)	7 (100.0)	--

*Overall data includes four patients who underwent a lung transplant.

Table S2. Overall time intervals in the study cohort and differences in time intervals between patients who recovered vs. died while waiting for a lung transplant.

**Time intervals (median [IQR])**	**Overall**	**Recovered while awaiting transplant**	**Died while awaiting transplant**	**P value**
Time from COVID-19 infection to hospital admission	4.00 [0.00, 7.25]	4.00 [0.00, 7.00]	5.00 [0.50, 10.50]	0.384
Time from COVID-19 infection to ICU admission	10.00 [5.75, 14.25]	9.00 [6.00, 15.00]	12.00 [4.00, 16.50]	0.958
Time from COVID-19 infection to MV	11.00 [7.75, 15.25]	10.00 [9.00, 15.00]	12.00 [4.00, 21.00]	0.873
Time from COVID-19 infection to ECMO	15.00 [11.50, 24.50]	15.00 [13.00, 30.00]	12.00 [7.00, 26.00]	0.289
Time from COVID-19 infection to transfer from another hospital	97.50 [59.75, 130.00]	73.50 [58.75, 109.25]	89.00 [61.50, 124.75]	0.999
Time from COVID-19 infection to listing	89.00 [65.75, 140.25]	87.00 [65.00, 98.00]	79.00 [52.50, 116.00]	0.672
Time from COVID-19 infection to death	117 [107.00, 169.50]	--	115.00 [106.00, 147.50]	--
Time from MV to ECMO	3.50 [1.25, 11.75]	3.50 [0.00, 29.75]	3.00 [2.00, 4.50]	0.77
Time from ECMO to listing	66.50 [38.00, 112.25]	56.00 [32.00, 68.00]	68.00 [44.00, 103.00]	0.368
Time from ECMO to death	101.00 [89.25, 156.25]	--	99.00 [83.50, 137.50]	--

Time from hospital admission to ECMO	10.00 [5.75, 17.00]	9.00 [7.00, 30.00]	5.00 [4.00, 13.00]	0.137
Time from hospital admission to MV	5.50 [3.00, 10.00]	7.00 [5.00, 9.00]	3.00 [1.00, 6.00]	0.11
Time from admission to listing	85.50 [65.75, 140.25]	80.00 [65.00, 98.00]	74.00 [47.50, 116.00]	0.711
Time from hospital admission to death	112.00 [100.25, 168.75]	--	106.00 [97.50, 147.50]	--
Hospital admission time	171.50 [105.75, 258.50]	226.00 [135.00, 302.00]	106.00 [97.50, 147.50]	0.017
ICU admission time	110.00 [90.00, 166.50]	117.00 [84.00, 139.00]	101.00 [94.50, 140.00]	0.958
Time on MV	122.50 [79.25, 178.50]	142.00 [58.00, 222.00]	101.00 [90.00, 140.00]	0.958
Time on ECMO	94.00 [58.50, 126.50]	59.00 [53.00, 93.00]	99.00 [83.50, 137.50]	0.044
Time on Tx list	20.50 [14.50, 29.25]	20.00 [16.00, 25.00]	29.00 [21.00, 53.00]	0.186
